# Hybrid BCI for Meal-Assist Robot Using Dry-Type EEG and Pupillary Light Reflex

**DOI:** 10.3390/biomimetics10020118

**Published:** 2025-02-18

**Authors:** Jihyeon Ha, Sangin Park, Yaeeun Han, Laehyun Kim

**Affiliations:** 1 Bionics Research Center, Korea Institute of Science and Technology, Seoul 02792, Republic of Korea; hj910410@kist.re.kr (J.H.); hye1230@kist.re.kr (Y.H.); 2 Next-Generation Mechanical Design Laboratory, Korea University, Seoul 02841, Republic of Korea; sipark84@korea.ac.kr; 3 Department of HY-KIST Bio-Convergence, Hanyang University, Seoul 04763, Republic of Korea

**Keywords:** electroencephalography (EEG), brain–computer interface (BCI), meal-assist robot, dry-type EEG, flash visual evoked potential (FVEP), eyewear-type infrared cameras, pupillary light reflex (PLR), electromyogram (EMG)

## Abstract

Brain–computer interface (BCI)-based assistive technologies enable intuitive and efficient user interaction, significantly enhancing the independence and quality of life of elderly and disabled individuals. Although existing wet EEG-based systems report high accuracy, they suffer from limited practicality. This study presents a hybrid BCI system combining dry-type EEG-based flash visual-evoked potentials (FVEP) and pupillary light reflex (PLR) designed to control an LED-based meal-assist robot. The hybrid system integrates dry-type EEG and eyewear-type infrared cameras, addressing the preparation challenges of wet electrodes, while maintaining practical usability and high classification performance. Offline experiments demonstrated an average accuracy of 88.59% and an information transfer rate (ITR) of 18.23 bit/min across the four target classifications. Real-time implementation uses PLR triggers to initiate the meal cycle and EMG triggers to detect chewing, indicating the completion of the cycle. These features allow intuitive and efficient operation of the meal-assist robot. This study advances the BCI-based assistive technologies by introducing a hybrid system optimized for real-world applications. The successful integration of the FVEP and PLR in a meal-assisted robot demonstrates the potential for robust and user-friendly solutions that empower the users with autonomy and dignity in their daily activities.

## 1. Introduction

Assistive technologies, such as meal-assist robots, play an important role in improving the quality of life of elderly and disabled populations [1]. Despite their potential, traditional meal-assisted robots often face challenges in terms of usability and adaptability, particularly for users requiring intuitive and efficient interactions [2]. Brain–computer interfaces (BCIs), which enable direct communication between the brain and external devices using noninvasive electroencephalograms (EEGs), have emerged as a promising solution to this challenge [3,4].

BCIs utilize various modalities to interpret the user intent. Representative approaches include motor imagery (MI), event-related potentials (ERPs), steady-state visual evoked potentials (SSVEP), and flash visual evoked potentials (FVEP). MI-based BCIs enable control through imagined hand movements, offering intuitive interaction, but require significant user training [5,6]. ERP-based systems rely on brain responses to specific stimuli, providing reliable performance, but demanding repetitive engagement, which may reduce practicality [7,8]. SSVEP-based BCIs detect brain responses to periodic visual stimuli at fixed frequencies, delivering high classification accuracy, fast information transfer rates (ITR), and requiring minimal user training, making them particularly effective in assistive applications [9,10]. An FVEP-based system [11] utilizes brain responses induced by rapid light stimuli. It is typically simpler in design and faster in response than ERP-based BCIs, while being less robust to noise than SSVEP-based BCIs.

In our previous study, we developed an SSVEP-based BCI system utilizing wet-type EEG for meal-assisted robot control, achieving an average accuracy of 83.33% and an ITR of 20.41 bit/min [12]. Wet-type EEGs are known for high signal quality and reliability in BCI systems. However, their dependence on conductive gels introduces practical challenges including lengthy preparation times, discomfort during prolonged use, and potential signal degradation as the gel dries over time. These limitations make wet-type EEGs less suitable for real-world user-centric applications, such as assistive robotics, where ease of use and comfort are critical.

By contrast, dry-type EEGs eliminate the need for conductive gels and offer the potential for a quicker setup and improved usability [13]. However, they face challenges such as low signal quality, susceptibility to motion artifacts, and variability in the electrode–skin contact impedance. Recent advancements in dry electrode technology have sought to address these issues by exploring novel materials [14,15], improved signal acquisition techniques, and advanced artifact reduction algorithms [13].

Many studies have extensively explored the integration of additional modalities with EEGs to enhance the BCI performance. Many of these studies focused on wet-type EEG; however, notable examples include combining SSVEP with electrooculogram (EOG) [11] or integrating SSVEP with eye-tracker systems [16]. In this study, to further improve usability and address the inherent limitations of dry-type EEG, the incorporation of additional modalities, such as the pupillary light reflex (PLR), has emerged as a hybrid approach. The PLR, which is the automatic constriction and dilation of pupils in response to light, has recently gained recognition as a viable input modality for BCIs [17]. Moreover, the PLR is non-invasive and naturally elicited, making it particularly suitable for real-world applications. Several studies demonstrated that the PLR can effectively differentiate between multiple visual stimuli with high accuracy [17,18].

In this study, we propose a novel hybrid BCI system that combines a dry-type EEG for FVEP and eyewear-type infrared cameras for PLR, specifically designed for integration into a hardware LED-based meal-assist robot system. We hypothesized that FVEP and PLR would both be elicited in response to four types of LED stimuli blinking at different frequencies and that utilizing both responses together would achieve higher performance. The primary contribution of this study is a hybrid BCI system that integrates FVEP and PLR to achieve acceptable levels of accuracy and ITR. The use of dry-type EEG and eyewear-type infrared cameras further enhances the usability by simplifying the setup process and improving user comfort.

This study compared and validated the proposed hybrid BCI system through three experimental stages. The first experiment used flickering hardware LEDs for the wet-type EEG-based SSVEP. The second experiment used an eyewear-type infrared camera-based PLR. The final experiment was a hybrid approach using a dry-type EEG-based FVEP and eyewear-type infrared-camera-based PLR. Derived from the experiments, we implemented a validated hybrid BCI system in a real-time meal-assisted robot control scenario.

## 2. Materials and Method

### 2.1. Off-Line System

#### 2.1.1. System Configuration

The system used a laptop with an Intel CPU i9-13980HX 2.20 GHz, RAM 64 GB to measure the EEG and pupil size. We used an Arduino Uno to control the four LEDs. A laptop and an LED controller communicated via serial communication. EEG of 64 channels using wet-type electrodes was measured using Active Two (Biosemi S. V., Amsterdam, The Netherlands) at a sampling rate of 2048 Hz, while 20 channels EEG using dry-type electrodes was measured using the Quick-20 system (Cognionics Inc., San Diego, CA, USA) at a sampling rate of 500 Hz. The data acquisition software was a lab streaming layer (LSL) [19] and Python 3.10 (Python Software Foundation, Nework, DE, USA). Binocular pupil images were captured using a Pupil Core eye tracker (Pupil Labs, Berlin, Germany) with a frame rate of 30 Hz and an image resolution of 192 × 192 pixels and Pupil Capture software (v3.5.1.). The Pupil Lab cameras were mounted on custom-designed glasses-type devices, referred to as eyewear-type infrared cameras.

#### 2.1.2. Experimental Environment and Design

**Figure 1:**
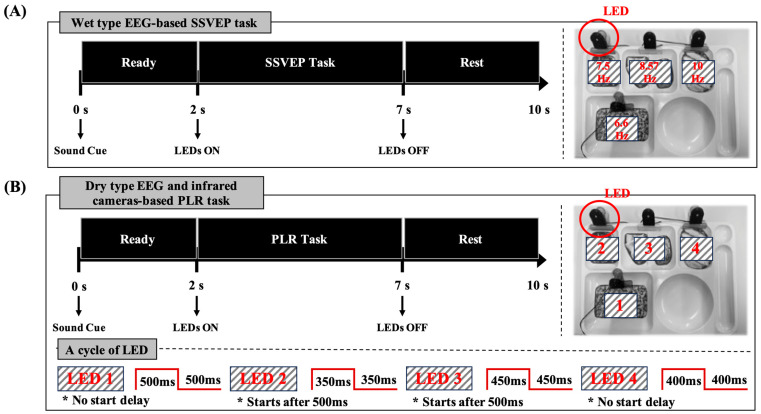
Overview of experimental design and procedure. (**A**) Wet-type EEG-based experiment. (**B**) Infrared camera-based experiment and dry-type EEG with infrared camera-based experiment. “* No start delay” indicates that LED1 and LED4 begin flashing immediately without any initial delay. “* Starts after 500 ms” indicates that LED2 and LED3 begin flashing 500 ms after the initial start time.

This study consisted of three stages. The first stage was a wet EEG-based SSVEP experiment. This experiment aims to evaluate and compare the accuracy of conventional SSVEP-based BCI systems. Four flickering LEDs were utilized, operating at frequencies of 6.6 Hz (LED 1), 7.5 Hz (LED 2), 8.57 Hz (LED 3), and 10 Hz (LED 4), each flickering for 5 s [11]. During a trial, participants listened to a sound cue (“One”, “Two”, “Three”, or “Four”) and focused on the corresponding LED within 2 s. They then gazed at the flickering LED for 5 s, followed by a 3 s rest period. Each trial was repeated 60 times, with the LEDs presented in a random order. The experimental setup and procedure for this stage are shown in Figure 1A.

The second stage involved an eyewear-type infrared camera-based PLR experiment. Although the experimental process was identical to that in the first stage, the flickering rules for the LEDs differed. The LEDs flickered according to the following patterns, each lasting 5 s: (1) LED 1 flickered immediately with no delay, alternating between 500 ms on and 500 ms off. (2) LED 2 started flickering with a 500 ms delay, alternating between 350 ms on and 350 ms off, and turned off completely after 2500 ms. (3) LED 3 began flickering after a 500 ms delay, alternating between 450 ms on and 450 ms off. (4) LED 4 flickered immediately with no delay, alternating between 400 ms on and 400 ms off, and turned off completely after 2500 ms. The experimental process for this stage is illustrated in Figure 1B.

In the third stage, a hybrid experiment was conducted using dry-type EEG-based FVEP and eyewear-type infrared-camera-based PLR. The procedure and LED flickering rules are identical to those used in the second stage. Thus, the experimental setup and process for this stage are the same as the second stage, shown in Figure 1B.

**Figure 2:**
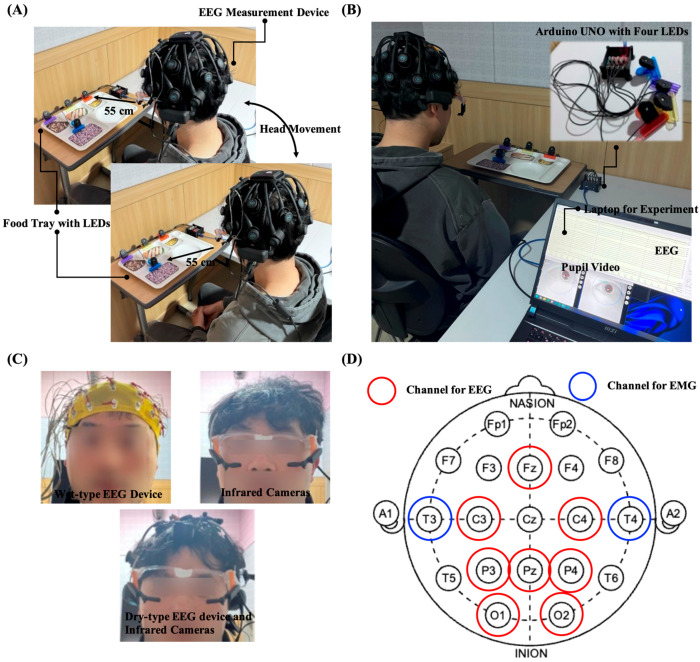
Overview of the experimental environment. (**A**) LEDs on the food tray and the participant. (**B**) Experimental laptop and Arduino UNO based LED system. (**C**) Participant wearing the equipment used in each experiment. (**D**) Experimental monitoring system.

The distance between the participant’s eyes and LEDs was set to 55 cm. Because the distance between LED 1 and LED 2–4 was different, the participants were instructed to move their head slightly during the experiment to maintain a distance of 55 cm. The equipment used, the experimental environment, and the hardware settings for all the stages are shown in Figure 2.

#### 2.1.3. Participants

Five participants, including two males and three females (mean age: 30.4 ± 3.05 years), were recruited for this study. All participants had prior experience with SSVEP experiments using liquid-crystal-display-based systems. After completion of the three experimental sessions, monetary compensation was provided to the participants. This study was reviewed and approved by the Institutional Review Board of the Korea Institute of Science and Technology Institutional Review Board (IRB approval number: KIST-202405-HR-001).

#### 2.1.4. Data Acquisition and Analysis

##### Wet-Type EEG-Based SSVEP Experiment

For the SSVEP feature extraction, three channels (O1, Oz, and O2) in the occipital region were selected from the 10–20 system. Signal processing and SSVEP analysis were conducted using MATLAB 2023a (MathWorks, Inc., Natick, MA, USA). Data were referenced by subtracting the Cz signal. An elliptical IIR bandpass filter was then applied with cutoff frequencies set at 2 Hz (low) and 54 Hz (high). The SSVEP was detected using an extension of the multivariate synchronization index (EMSI) [20]. The performance of SSVEP was verified by calculating its accuracy (%) and ITR (bit/min). Detailed descriptions and equations for the ITR are as follows:

$$B\;\left(\frac{Bit}{Trial}\right)=\log_{2}N+P\times \log_{2}P+\left(1-P\right)\times \log_{2}\left(\frac{1-P}{N-1}\right)$$

$$Q\left(\frac{Trials}{Min}\right)=\frac{S}{T}$$

$$ITR\left(\frac{Bit}{Min}\right)=B\times Q$$

where *B* denotes the information transferred in bits per trial, *N* represents the number of targets, *P* indicates the classification accuracy, and *Q* denotes the average classification time in min.

##### Eyewear-Type Infrared Camera-Based PLR Experiment

**Figure 3:**
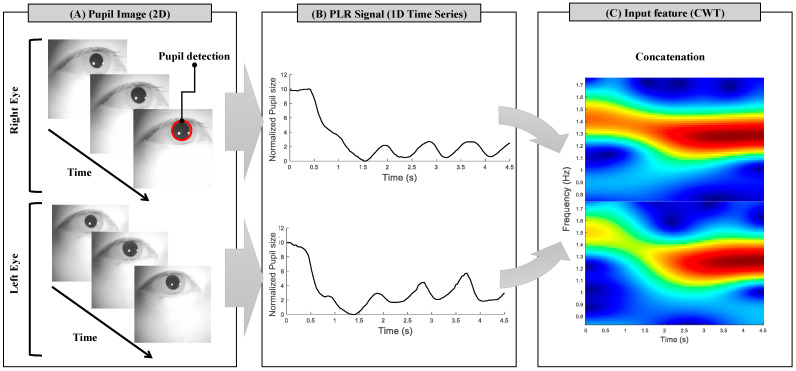
Schematic of the pupillary light reflex (PLR) feature extraction procedure. (**A**) The red circle indicates the estimated pupil detection from grayscale images of binocular eyes. (**B**) The time-series PLR signals are measured during the experiments. The *y*-axis represents the normalized pupil size. (**C**) The input features for classification are extracted using continuous wavelet transforms (CWTs) from the time-series PLR signals.

For PLR feature extraction, binocular pupil images were acquired using a Pupil Core eye tracker with a frame rate of 30 Hz and an image resolution of 192 × 192 pixels. For the automatic pupil detection, pupil images were processed using a pupil detection algorithm and analyzed while running the Pupil Capture software v634d13a [21]. The image processing steps are as follows: (1) The images were first converted into grayscale. (2) Within the images, the region of the pupil was initially estimated using a previously proposed method based on a Haar-like feature detector [22]. (3) The potential pupil area, divided into sub-contours, was detected using Canny edge detection and histogram analysis. (4) The detected pupil edge was fitted to an ellipse, and the confidence value associated with the ellipse was calculated. (5) If the confidence value was higher than the predefined threshold, the candidate ellipse was marked as the detected pupil [21]. Step A of Figure 3 shows the estimated pupil region detected through these steps, highlighted with a red circle. After pupil detection from the images, the three-dimensional pupil size was converted to a CSV file and used for analysis. To synchronize the pupil data with the timing of the visual stimuli triggers, serial communication was used between laptops. The time-series PLR signals for both eyes were stored as pupil data and used for PLR feature extraction, as shown in step B of Figure 3. Signal processing and analysis of the pupil data were conducted using MATLAB v2021.

Then, we visually inspected and rejected invalid trials (eye blinks and artifact detection) for each eye during the task. For the classification of PLR patterns according to the light stimulation of the four LEDs, the spectral-temporal PLR features were derived from pupil data with a length of 4.5 s. Continuous wavelet transforms (CWTs) were applied to extract features. The analysis employed a Morse wavelet as the mother wavelet with default parameters ($\gamma =3,p^{2}=60$) [23]. The binocular spectral–temporal PLR features were extracted as time-series wavelet power spectra within the frequency range of 0.5–1.75 Hz, considering the frequency of LED 2 with the shortest flickering cycle in both the PLR and hybrid experiment, i.e., as depicted in step C of Figure 3. For a detailed description and figure of the PLR features based on the CWT method, refer to Supplementary Materials (Figure S1).

**Figure 4:**
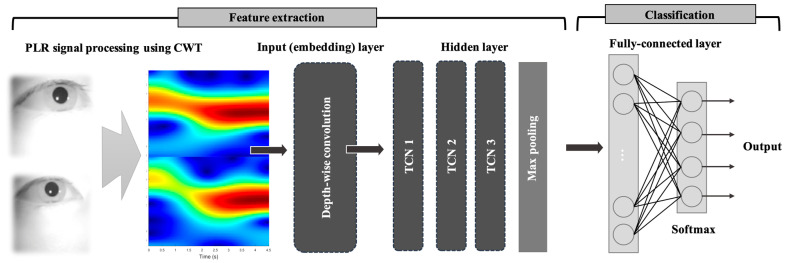
Deep leering architecture for PLR classification.

The CWT-based binocular PLR features are passed into an embedding layer to extract initial features from the input data. The output features from the embedding layer are processed through multiple 1D temporal convolution network (1D TCN) layers to capture precise temporal patterns [24]. Table 1 shows parameters of the 1D-TCN model. Next, a max pooling layer reduces the feature map size while retaining important information. The processed features are then sent to a fully connected layer and an output layer for final classification. Center loss is applied to the features before the softmax activation function to make the output more discriminative [25]. The detailed procedure of the PLR feature extraction and deep learning classification using PLR features are illustrated in Figure 3 and Figure 4, respectively.

PLR classification performance was evaluated using leave-one-subject-out cross-validation (LOSOCV), with each fold consisting of single-subject data. The classification accuracy and ITR of the validation set were used to evaluate and compare the performances of the four PLR pattern classification models.

Dry-type EEG-based FVEP in the hybrid experiment. For the FVEP feature extraction, three channels (Pz, O1, and O2) in the parietal–occipital regions were selected from a 10–20 system in a dry-type EEG headset. The 3rd order of Butterworth (IIR) band-pass filter was applied with cutoff frequencies of 2 and 30 Hz.

The FVEP represents stimulus-locked neural activity that is synchronized with the timing of flash onset (or offset) and has neural components of within 200 ms after flash stimulus. Therefore, unlike the SSVEP classification method, FVEP uses a time-domain analysis approach. However, it is difficult to pre-segment EEG data into a fixed time window length considering that there are four different flickering cycles. Thus, the EEG data were segmented at each timing, from 100 ms before to 350 ms after, based on the flickering rules of target and non-target LEDs that were set in the hybrid experiment. The segmented epochs of flash onset and offset were averaged (referring to averaged onset FVEP and averaged offset FVEP), and the extracted features used peak-to-valley amplitudes [11]. Each trial consisted of four features, with each feature corresponding to the flickering timing of LED1 to LED4. To determine which LED is the target stimulus, we constructed an individual binary classification model for each LED. During classification, each model outputs whether the feature generated from the given LED flickering cycle corresponds to the target stimulus. The final decision on which LED was the target was based on the combination of predictions from all four models. This approach ensures that each feature is analyzed independently for target relevance, by focusing on specific timing information for each LED flickering cycle. We adopted support vector machine (SVM) as classifier and used grid search for SVM optimization [26].

For validation procedures, the performance of the FVEP classification was evaluated using the repeated hold-out method with a subject-dependent classification model due to inter-participant variability. A three-to-seven ratio of data was allocated to validation and training sets, respectively, and the holdout validation procedure was repeated 30 times [27,28]. The classification accuracy and ITR of the validation set were used to assess the performances of the FVEP classification model.

##### Hybrid Experiment: Dry-Type EEG-Based FVEP and Eyewear-Type Infrared Camera-Based PLR

For the hybrid BCI, eight channels (Fz, Pz, P4, P3, O1, O2, C3, and C4) were selected from a 10–20 system in a dry-type EEG headset. The 3rd order of Butterworth (IIR) bandpass filter was applied with cutoff frequencies of 0.7 and 54 Hz. In this study, a multichannel EEG was processed into a covariance matrix, defined as a subspace of the Euclidean space [29], which was used to detect the onset of stimuli. In the framework of EEG, X ∈ R C×N denote a segmentation of an EEG signal, where C is the number of channels and N is temporal samples. The covariance matrix was estimated as follows:

$$\Sigma =\frac{1}{N-1}XX^{T}.$$

To capture the temporal dynamics of brain states, the dynamic sliding window method was applied to the local covariance matrix estimation. This was implemented using the global field power (GFP) of the EEG to determine the sliding window sample points within the microstates. GFP is the global strength of the scalp electrical fields, which was calculated as follows [30]:

$$G\left(t\right)=\sqrt{{\frac{\left[\Sigma_{i}^{k}(V_{i}\left(t\right)-V_{mean}\left(t\right))\right]}{C}}^{2}},$$

where t is the time, C is the number of EEG channels, $V_{i}$ is the voltage in channel i, and $V_{mean}$ is the mean of the voltages in all channels. The local troughs of GFP were extracted, and then the covariance matrix Σ within a specific window length based on the troughs was calculated [31]. The temporal covariance matrices are symmetric and positive definite (SPD) and can be processed in a Riemannian manifold M with dimensions m = C(C + 1)/2: Based on the Riemannian framework, the affine invariant Riemannian (AIR) distance method was used to detect brain-state transitions by calculating the distance difference between two temporal covariance matrices [32]. For the computation of Riemannian distance between the reference covariance matrix ($\Sigma_{Ref})$ and the covariance matrix at a specific time point ($\Sigma_{Target})$, 3 pairs of EEG epochs (${Epoch}_{Ref},\;{Epoch}_{Target})$ were utilized in the following time range: Pair 1 (${Epoch}_{\left[-4,-3\right]},\;{Epoch}_{\left[-3,\;0\right]}$), Pair 2 (${Epoch}_{\left[-3,\;0\right]},\;{Epoch}_{\left[0,\;5\right]}$), Pair 3 (${Epoch}_{\left[0,\;5\right]},\;{Epoch}_{\left[5,\;8\right]}$). Time 0 represents the stimulus onset time. For a comprehensive description of the methods and a related figure on the Riemannian distance analysis of EEG data, refer to the Supplementary Materials (Figure S2).

**Figure 5:**
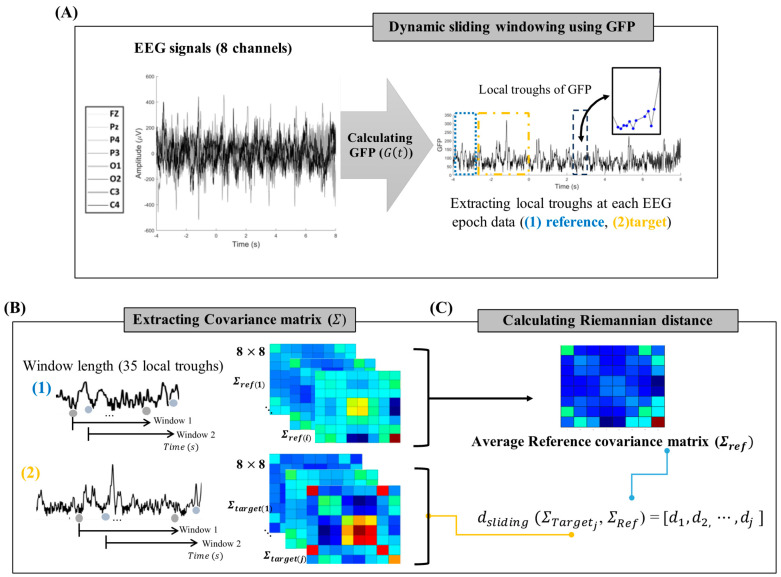
Process involved in extracting the dynamic Riemannian distance (*d_sliding_*). (**A**) The local troughs were extracted from GFP of 8 channels (Fz, Pz, P4, P3, O1, O2, C3, and C4). The black box represents an example of local troughs in a specific window, while the blue and yellow boxes indicate the reference and target EEG segment data for calculating the Riemannian distance, respectively. (**B**) An SPD matrix was extracted for the 8 channels within time window length. Each EEG segment data corresponds to a time window containing 35 local troughs. The reference SPD matrices were constructed using EEG data preceding the current time point for distance calculation. (**C**) The target SPD matrix was aligned along the time series using a local troughs-based sliding window method. It then calculates the Riemannian distance from the averaged reference SPD matrix.

Each epoch produces multiple covariance matrices by applying the dynamic sliding-window method. In ${Epoch}_{Ref}$, eigenvalue decomposition was performed on the covariance matrices formed from the latter half of the epoch. The average of the five covariance matrices with the lowest eigenvalue-based entropy was then computed to extract a single $\Sigma_{Ref}$. The AIR distance between the $\Sigma_{Ref}$ and $\Sigma_{Target}$ was calculated as [33]:

$$d\;(\Sigma_{Target},\;\Sigma_{Ref})={\left\|Log\left({\Sigma_{Target}}^{{-}_{2}^{1}}\Sigma_{Ref}{\Sigma_{Target}}^{{-}_{2}^{1}}\right)\right\|}_{F}={\left(\sum_{c}^{C}{log}^{2}\lambda_{C}\right)}^{\frac{1}{2}},$$

$$d_{sliding}\;(\Sigma_{{Target}_{j}},\;\Sigma_{Ref})=[d_{1},\;d_{2},\;\cdots ,d_{j\;}],$$

where $\Sigma^{{-}_{2}^{1}}$ is the symmetric inverse square root and $\lambda_{C},\;c=1,\cdots$, C are the eigenvalues of ${\Sigma_{Target}}^{{-}_{2}^{1}}\Sigma_{Ref}{\Sigma_{Target}}^{{-}_{2}^{1}}$. $d_{sliding}$ denotes the AIR distance within the sliding window, and j refers to the local trough points of the GFP at ${Epoch}_{Target}.$ The detailed process is illustrated in Figure 5.

**Figure 6:**
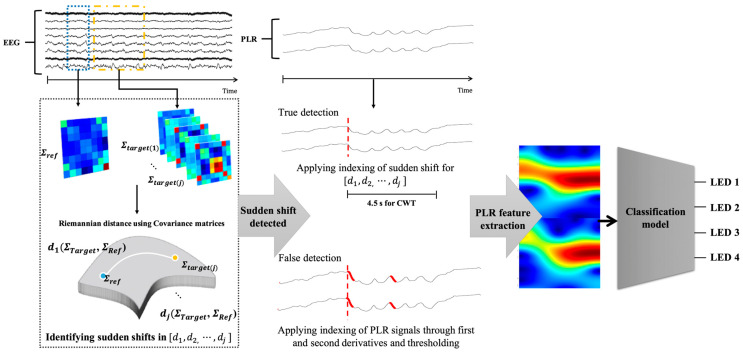
Conceptual diagram of the proposed dry-type EEG-based Hybrid BCI system.

After calculating the Riemannian distance over time, if sudden shifts in distance were detected, this transition was identified as the perception of the flickering LED stimulus. Using this approach, the first and second derivatives of the Riemannian distance signals were calculated. By setting thresholds at three and four times the standard deviation of each derivative, points with significant changes in the Riemannian distance were identified and used as indices for stimulus onset. If the difference in Riemannian distance values within the three troughs before and after the index was not less than 0.1, the brain state was deemed unrelated to the response to the LED stimulus. In such cases, PLR detection was performed to apply a secondary index adjustment. This involved re-epoching the EEG data starting from the index detected through the Riemannian distance calculation. If the Riemannian distance did not detect sudden shifts, the trial was excluded, similar to the valid trial rejection in the PLR experiment. The detailed process is illustrated in Figure 6.

The performance of the hybrid BCI classification was evaluated using leave-one-subject-out cross-validation (LOSOCV), with each fold consisting of single subject data. The classification accuracy and ITR of the validation set were used to evaluate and compare the performances of the four PLR pattern classification models.

### 2.2. Real-Time System with Meal-Assist Robot

#### 2.2.1. System Configuration

**Figure 7:**
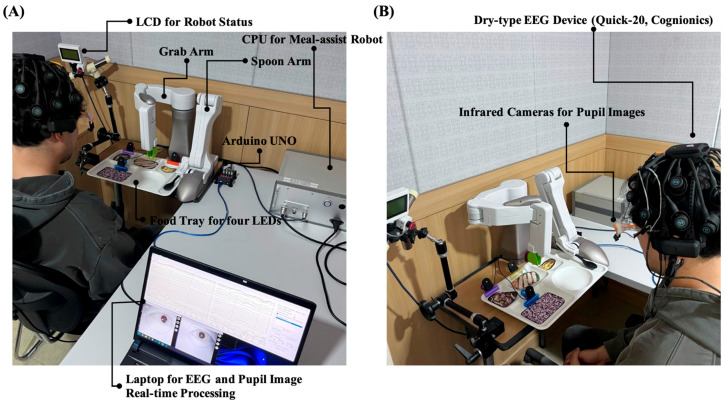
Devices for real-time system. (**A**) Components of meal-assist robot, Arduino UNO for controlling LEDs and laptop. (**B**) Devices for EEG and pupil images acquisition.

The proposed system used a laptop with an Intel CPU i9-13980HX 2.20 GHz, RAM 64 GB) to measure the EEG with pupil size and control the meal-assist robot. We used an Arduino Uno to control the four LEDs. A laptop and an LED controller communicated via serial communication. The meal-assist robot, “Caremeal”, used in this study was manufactured by NT robot (2004; Seoul, Republic of Korea) [34]. The “Caremeal” comprises a grab (5-axis motor) arm and a spoon (2-axis motor). The laptop and meal-assist robot communicated over Bluetooth 2.0 version (2.402–2.480 GHz). Twenty-channel EEG with dry-type electrodes was measured using a Quick-20 system with a sampling rate of 500 Hz. For real-time data acquisition, we used LSL for EEG and Pupil Capture software in Python 3.10. The hardware settings for the real-time system are shown in Figure 7.

#### 2.2.2. Proposed Triggers for Hybrid BCI System: PLR and Electromyogram (EMG)

**Figure 8:**
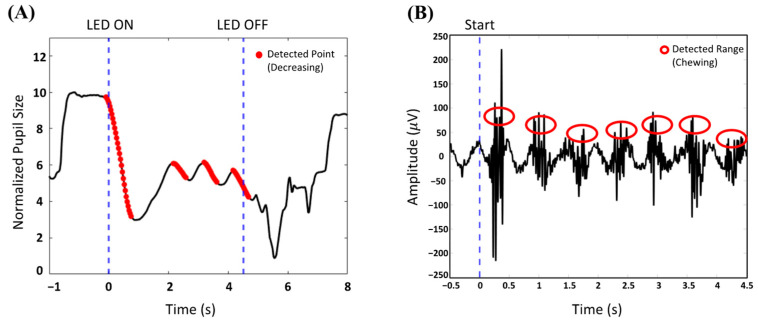
Participant 1 pupillary light reflex (PLR) and electromyogram (EMG). (**A**) Before and after PLR when the user gazes at the LED 4. (**B**) EMG when the user chews morsel.

In previous BCI-based meal-assisted robot studies, cycle initiation relied on three eyeblinks [12]. However, in this study, the system begins when the user gazes at a continuously flickering LED and PLR changes are detected. As shown in Figure 7, LED 4 was positioned outside the participants’ typical line of sight while seated in front of the meal-assisted robot and tray to ensure that the LED light did not interfere with their normal field of view. Before LED stimulation begins, or when the cycle is not intentionally activated, no significant PLR changes should occur for LED 4. An example of the change in pupil size before and after LED stimulation is shown in Figure 8A.

In this system, PLR and EMG do not function as continuously monitored signals but instead serve as event-based triggers to facilitate meal assistance. PLR detection is used only before meal initiation, where the system monitors the user’s gaze fixation on LED 4. Once a significant PLR change has been identified, the system triggers the start of the meal cycle, and further PLR monitoring is no longer required. After the grab arm places the selected food on the spoon arm, EMG monitoring becomes active to detect chewing activity. The system then identifies the onset of chewing through EMG from the temporal region (T3, T4), confirming that the user has taken a bite. An example of EMG is illustrated in Figure 8B. The EMG classification method used in this study followed the same approach as that used in our previous BCI-based meal-assist robot study [12], which achieved an accuracy of 97.33%. This detection serves as a trigger for the spoon arm to return to its original position, preparing the system for the next feeding action. Further details on the real-time operation of these triggers are provided in Section 3.2.

### 2.3. Statistical Analysis

We performed the statistical analyses on the EEG and PLR related features used in the hybrid method. First, the statistical differences between the CWT-based PLR features of each LED stimuli across all participants were assessed by using a permutational multivariate analysis of variance (PERMANOVA) for the non-parametric statistical tests [35,36]. Second, we verified the statistical significance of the difference in the average dynamic Riemannian distances between 0.5 s before and after the onset of the LED stimuli. A Wilcoxon signed rank test was used, since the normal distribution was not met. The PERMANOVA was performed with 1000 permutations, and all statistical analyses were conducted in RStudio (Version: 2024.12.0+467).

## 3. Results

### 3.1. Experimental Result for Off-Line System

**Figure 9:**
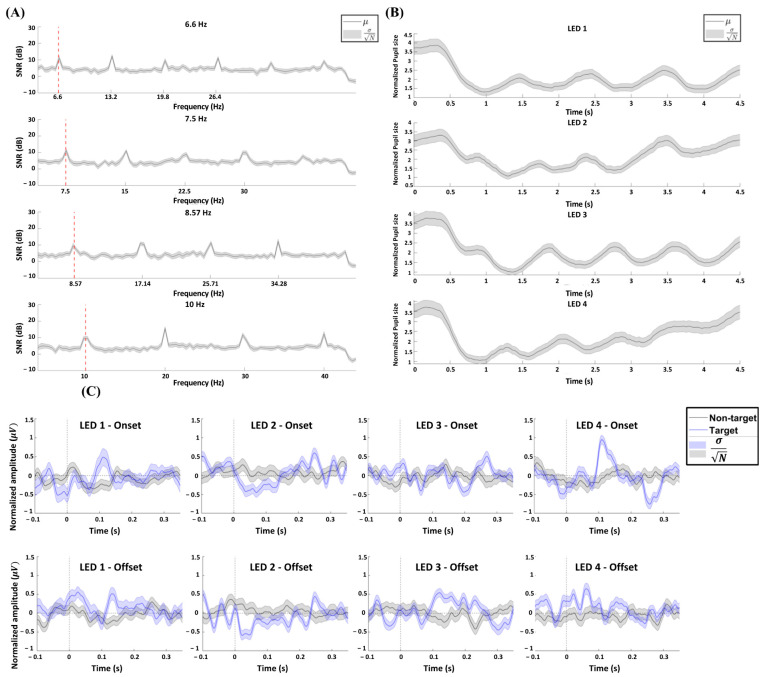
Averaged SSVEP, PLR, and FVEP from all participants. (**A**) Averaged SSVEP spectrum of the signal recorded from Oz electrodes for the four types of stimuli. The red dashed lines denote the fundamental frequencies with respect to target frequency. (**B**) Averaged PLR for the four types of luminance modulation patterns over 4.5 s. (**C**) Averaged FVEP onset and offset signals recorded from O1, O2, and Pz for target and non-target trials for each of the four types of stimuli.

Figure 9 illustrates the responses of each modality when watching the targets. Figure 9A shows the average SSVEP spectrum in the Oz channel acquired during the wet-type EEG-based experiment. The plot represents the average signal-to-noise ratio (SNR) between 0 and 4.5 s, relative to the stimulus onset. Figure 9B shows the average PLR time-series patterns acquired during the eyewear-type infrared camera-based experiment. Similarly, the plot shows the average PLR from 0 to 4.5 s relative to stimulus onset. Figure 9C shows the average FVEP onset and offset signals time-series patterns in the O1, O2, and Pz channels acquired during the hybrid experiment. The blue and black lines represent target and non-target trials, respectively, for each of the four types of stimuli. The plot represents the time-series average EEG from −0.1 s to 0.35 s relative to the stimulus onset and offset.

**Figure 10:**
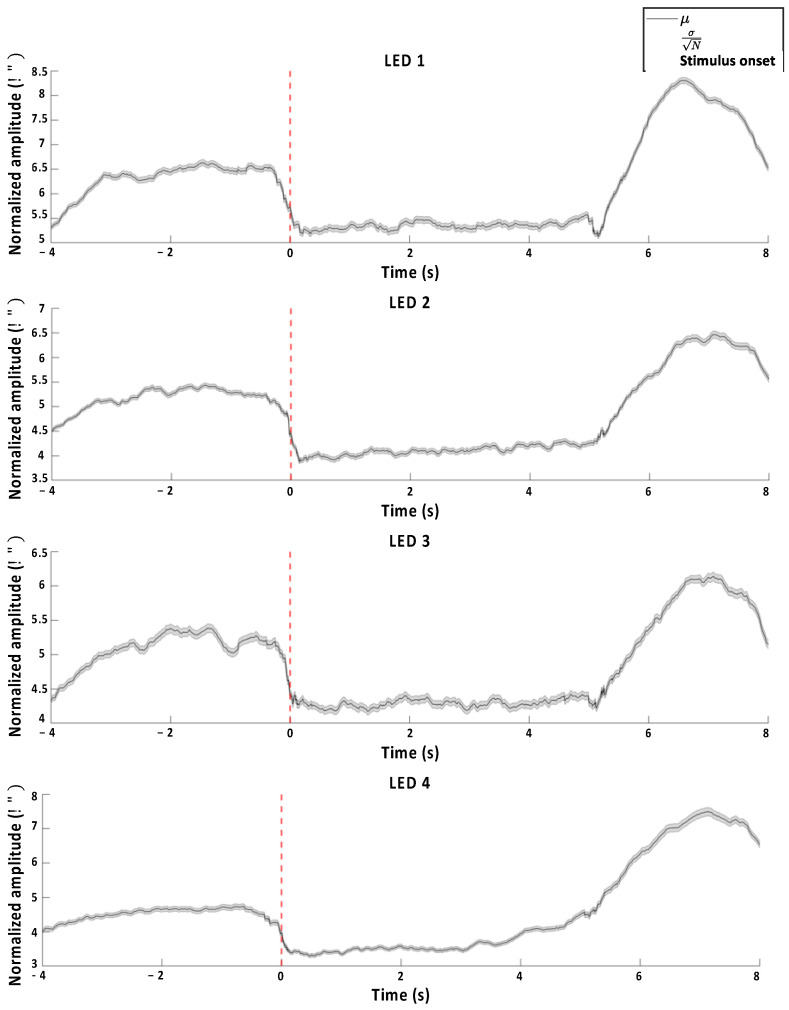
Target-wise average dynamic Riemannian distance across all subjects from the hybrid experiment.

Figure 10 illustrates the dynamic Riemannian distances averaged across all the subjects for each target class during the hybrid experiment. A statistically significant difference was found in the average dynamic Riemannian distance between pre- and post-LED stimulus onset (*p* < 0.001, Wilcoxon signed test).

**Figure 11:**
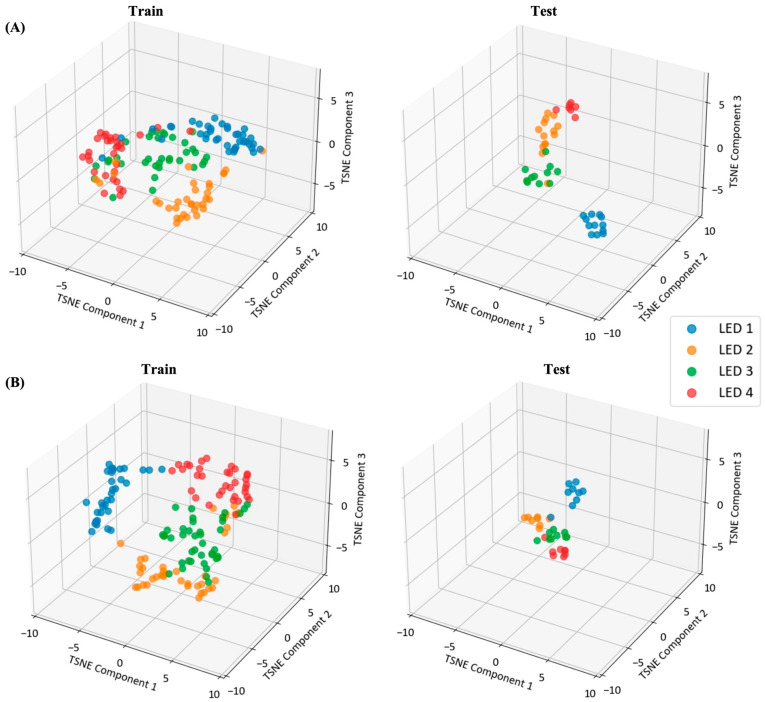
1D-TCN-based feature visualization of using t-distributed stochastic neighbor embedding (t-SNE) at different systems (**A**) PLR features in the hybrid experiment, (**B**) PLR features in eyewear-type infrared camera-based PLR experiment.

Figure 11 shows the feature clustering of different systems (hybrid and PLR) using the t-distributed stochastic neighbor embedding (t-SNE) technique. The features extracted from the training set are projected onto a lower-dimensional space for clustering. Both systems effectively demonstrated the distribution of the feature space, and the hybrid BCI using dynamic indices exhibiting well-trained PLR-related feature clustering. The LED stimuli differed significantly from each other with respect to the CWT-based PLR features (PERMANOVA, df = 3, pseudo-F = 2.356, *p* = 0.014).

In this study, while the experiments lasted 5 s, data from 0 to 4.5 s were used for classification. Thus, the total time used for ITR calculation was 4.5 s. For FVEP classification, however, the entire 5 s were used in ITR calculation. For the first experiment, the wet-type EEG-based SSVEP analysis utilized three occipital channels: O1, Oz, and O2. The average accuracy and ITR of the five participants were 93.33% and 21.16 bit/min, respectively. In the second experiment, the infrared-camera-based PLR analysis achieved an average LOSOCV accuracy of 84.87% and an average ITR of 15.99 bit/min across five participants. In the third experiment, dry-type EEG-based FVEP classification, the average accuracy and ITR of repeated hold-out validation were 77.59% and 10.57 bit/min, in turn, over the five participants. In the fourth experiment, which analyzed data from both the dry-type EEG and infrared cameras, the average LOSOCV accuracy and ITR for the five participants were 88.59% and 18.23 bit/min, respectively. The individual performance of the participants in each experiment is presented in Table 2.

### 3.2. Simulation of the Real-Time System with Meal-Assist Robot

**Figure 12:**
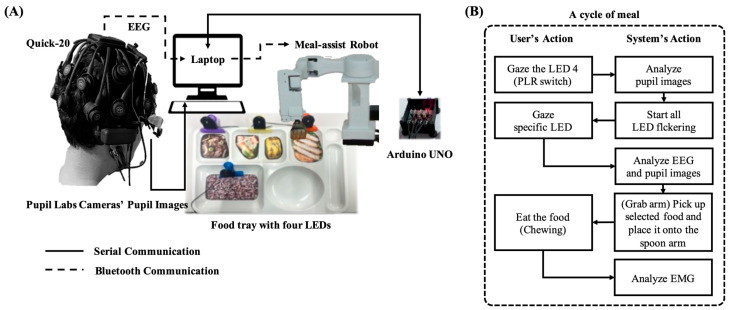
Schematic of the proposed interface-based meal-assist robot system. (**A**) Graphical flow chart for used devices. (**B**) Flow chart for the actual process.

In this study, we propose a meal-assisted robot control system based on user intention detection using a dry-type EEG device and eyewear-type infrared cameras integrated with an LED system. The system utilizes a PLR trigger to initiate a meal cycle and an EMG trigger to indicate the end of the cycle. A single meal cycle follows these steps: (1) The user gazes at the continuously lit LED 4 for more than 4.5 s, during which pupil changes are detected and LED 4 is turned off. (2) After 2 s, the four LEDs begin to flicker for 4.5 s, and the user looks at the LED corresponding to their desired food. (3) Based on the FVEP and PLR analyses, the grab arm moves to the selected food. (4) The grab arm picks up the morsel and places it on the spoon arm. (5) The spoon arm raises the morsel to a suitable height for the user approaching it to eat the morsel. (6) Once the user begins chewing, the spoon arm is lowered as detected by the EMG features. For safety, if no EMG is detected, the spoon arm automatically lowers after 10 s. (7) The meal-assisted robot system returns to a standby state and awaits the next meal cycle. In this system, the PLR trigger was active only during state (7), whereas the EMG trigger was active only during state (5). Figure 12A illustrates the overall process of the proposed system and Figure 12B shows the communication framework between the hardware components.

## 4. Discussion

The objective of this study was to propose a system that identifies user intent and controls a meal-assisted robot by utilizing a hybrid BCI based on mobile dry-type EEG-based FVEP and eyewear-type infrared camera-based PLR. We previously proposed an SSVEP BCI-based meal-assist robot system [12]; however, it relied on a wet-type EEG. In contrast, the proposed system uses a dry-type EEG, incorporating PLR to compensate for the lower accuracy of the dry-type EEG. Three experiments were conducted to evaluate the performance of the proposed hybrid BCI system. As shown in Figure 9, the experiments successfully elicited the SSVEP, PLR, and average FVEP responses. The wet-type EEG-based BCI experiment achieved the highest accuracy of 93.33% and an ITR of 21.16 bit/min. Even with PLR alone, the system achieved acceptable results with an accuracy of 86.36% and an ITR of 16.77 bit/min. The hybrid BCI experiment also demonstrated acceptable performance, with an accuracy of 88.59% and an ITR of 18.23 bit/min compared with PLR alone. Notably, the t-SNE patterns in Figure 11 show that both the PLR classification model and hybrid PLR-based classification model were well trained, even in subject-independent tests. This indicates that the proposed model, which is similar to the traditional SSVEP BCI systems, is highly practical because it does not require additional training.

This study makes four key contributions to the existing literature. First, this study proposes a Riemannian framework for dry-type EEG-based single-trial analysis. Figure 9C shows the average FVEP onset and offset signals for LED stimuli. This illustrates the difference between target and non-target trials in FVEP onset and offset patterns for the four types of flickering LED stimuli. However, a single-trial analysis is affected by a low SNR and the inherent variability of EEG signals. An average accuracy of 77.59% and an ITR of 10.57 bit/min were achieved across all subjects. However, this approach has limitations as it requires an individual training model for each subject and utilizes the entire 5 s EEG data to derive FVEP signals. To address this issue, previous studies used the Riemannian approach applied in the space of the covariance matrix to analyze sensor-based or source-based networks, classify multiple classes in various paradigms (MI, SSVEP, ERP, etc.), or improve machine-learning models [37,38,39]. Furthermore, Riemann-based machine learning outperformed a linear classification model using a single trial in MI-based BCI [40]. EEG is a multidimensional and complex neural activity of the brain, suggesting that the analysis of spatial EEG information is possible through various implementations of the nonlinear Riemannian manifold of the covariance matrix. Unlike the Euclidean space, the space of the covariance matrices is curved and flows along the surface of the matrix space without spatial constraints, which can provide a broader analysis of EEG signal processing [40].

In this study, we propose a Riemannian framework to detect the timing of task onset through the difference in neural activity from the pupil to the visual processing pathway when performing the task of looking at a flickering LED stimulus. This reflects the FVEP response at the onset of the LED stimulus and can be interpreted within the Riemannian framework as part of global brain neural activity, along with processes such as attention shifting. Dynamic covariance matrices and state-specific Riemannian distances were computed from the EEG time-series data. The differences in the Riemannian distances across the experimental stages are shown in Figure 10. One of the most interesting aspects is the observed reduction in the Riemannian distance from rest during the task. This is attributed to the sensitivity of the nonlinear Riemannian manifold to artifact detection and the analysis of complex multivariate time-series data based on cross-channel information or frequency components of EEG [41,42]. This suggests that the variability of neural activity in the brain at rest is reflected more significantly than the EEG patterns synchronized during the task. This approach enables the detection of significant changes in Riemannian distance based on EEG, which can be utilized as an index of task onset. In addition, this method can be applied to dry-type EEG-based asynchronous BCI systems.

Second, we proposed stimuli designed to simultaneously elicit FVEP and PLR. Recent advancements in dry-type EEG-based BCI research focused on improving the accuracy by developing new materials [14,43,44,45] or deep learning methods [13,46,47]. Unlike these approaches, this study not only introduced a novel method for utilizing dry-type EEG alongside the high-accuracy PLR modality [16] but also designed LED stimuli specifically to evoke both FVEP and PLR. The LED stimuli used in this study were closer to those optimized for PLR induction than the conventional SSVEP-induced LED stimuli, as they rely on FVEP triggered by light stimulation. The performance of a dry-type EEG-based SSVEP is generally lower than that of a wet EEG [13,48]. To address this limitation, we focused on analyzing FVEP rather than repetitive light stimulation. Consequently, the offset timing of the LED stimulus was not critical for the FVEP. Instead, varying the stimulus offset timing emphasizes the PLR changes for each LED. As a result, PLR alone achieved a strong performance, and combining it with EEG further improved the accuracy. Although the hybrid system outperformed the PLR in terms of performance, it also demonstrated significant practicality for real-world applications. In the PLR experiment, we corrected and analyzed only the trials in which it appeared that the participant had not directly gazed at the stimulus. However, this approach may result in greater performance degradation in real-world applications. In contrast, the proposed hybrid system automatically corrects cases in which the user is determined to have gazed at the LED using EEG, making the experimental results and real-world performance relatively robust.

Third, this study demonstrated that combining dry-type EEG electrodes with eyewear-type infrared cameras enables the intuitive use of triggers for meal-assisted robot operation. Our previous wet-type EEG-based meal-assist robot control system [12] initiated each meal cycle with three eye blinks, selected the desired food using SSVEP analysis, and detected chewing activity using EMG. Although the system is intuitive, the limitations of wet electrodes motivated this study. While it is possible to rely solely on PLR for ease of use and guaranteed accuracy, integrating a dry-type EEG allows the system to detect when a user chews food using an EMG. Additionally, unlike previous research, this study proposes initiating the meal cycle by gazing at a continuously lit LED 4, rather than using eyeblinks. Although triple blinking also achieves a very low false positive rate (FPR), directly gazing at the stimulus on the tray is simpler and more intuitive. Furthermore, the system utilizes the structural characteristics of the grab arm of the meal-assist robot to ensure that LED 4, which requires a slight head movement to view, does not interfere with the user’s vision. In real-world applications, the system ensures that only the PLR trigger is detected before the meal begins and the EMG trigger is only detected when the spoon arm lifts food to the user. This design minimizes the actual FPR.

Fourth, this study is not the first to propose a BCI-based meal-assist robot; however, it is the first to introduce a meal-assist robot using dry-type EEG and PLR. Previous studies [12,49,50] primarily utilized steady-state visual evoked potentials (SSVEP) with wet EEG electrodes for assistive robot control, achieving high classification accuracy but requiring conductive gel-based electrode application. While SSVEP offers robust signal quality with minimal training, wet-type EEG setups can be uncomfortable for long-term use and increase the setup complexity. In contrast, our system is designed to operate with dry electrodes, significantly improving user comfort and reducing preparation time. Beyond SSVEP-based systems, motor imagery (MI) and event-related potential (ERP) BCIs have also been explored for assistive applications. However, MI and ERP often require extensive user training and calibration to achieve reliable performance, making them less immediately accessible for new users [3,4]. Additionally, MI-based systems depend on voluntary motor cortex activation, which may not be feasible for all individuals with motor impairments. By integrating pupillary light reflex (PLR), our system provides a non-invasive, intuitive control method that does not require prolonged training or complex calibration. This unique combination allows users to engage with the meal-assist robot seamlessly, without the cognitive or physical demands associated with MI or ERP [5,6,7,8]. To further contextualize the performance of our approach, future studies will include direct comparisons with SSVEP-, MI-, and ERP-based assistive BCI systems. This will help evaluate the relative strengths and trade-offs in terms of usability, signal reliability, and user adaptability in real-world assistive applications.

This paper proposes a system that combines dry-type EEG and PLR in a controlled lighting environment. In future studies, we could explore various lighting conditions to determine the optimal scenarios for using the PLR alone. Additionally, in cases where ambient lighting significantly affects the PLR, the system can rely solely on the pupil position and dry-type EEG, enabling the hybrid interface to function effectively under any condition. Furthermore, previous studies improved the SSVEP classification accuracy by utilizing the minimum distance-to-mean method within the Riemannian framework, using cue onset time as a reference point [29]. In future work, we will consider further investigations to enhance the distance/divergence strategies within the Riemannian framework and reduce the FPR, enabling real-time and online experiments in asynchronous setups. Furthermore, we could explore hydrogel-based semi-dry electrodes as a potential solution to improve the signal quality of dry-type EEG and facilitate its practical use in BCI applications [14].

Beyond improvements in machine learning models, the accuracy and reliability of the system can also be enhanced by integrating context-aware intelligence into the assistive robot. For example, the system could incorporate adaptive learning mechanisms that account for user preferences and varying meal conditions. Since users may not always consume the same types of food, incorporating an adaptive correction mechanism that adjusts FVEP- and PLR-based predictions based on real-time feedback from the user could improve classification robustness. Additionally, integrating an intelligent decision-making framework that accounts for user hesitation or misclassification could further enhance the reliability of the system in practical applications.

In addition to these application-driven improvements, deep learning techniques have shown promising results in EEG signal processing, and could further enhance FVEP-based classification. Rather than relying on manually extracted features, end-to-end deep learning models can learn FVEP representations directly from raw EEG signals, improving feature extraction and classification accuracy. Convolutional neural networks have been successfully applied to extract spatial features from EEG signals [51], while recurrent architectures such as LSTMs [52] or transformers [53] have demonstrated their effectiveness in modeling temporal dependencies in brain signals. These approaches could allow for more robust and adaptive FVEP classification, potentially increasing system performance in diverse real-world scenarios. Future research will focus on integrating these deep learning methods to further optimize accuracy and usability in assistive robotics applications.

The proposed hybrid BCI system has the potential to enhance the accessibility and usability of meal-assist robots, particularly for individuals with upper limb impairments or those with severely limited arm mobility. Unlike traditional assistive feeding devices that often require manual adjustments or external assistance, this system allows users to interact with the robot using physiological signals, enabling a more autonomous and intuitive feeding experience. In real-world applications, users with restricted upper limb movement could initiate feeding actions using PLR-based selection, while EMG triggers would confirm chewing and progress the feeding cycle. This approach not only reduces reliance on caregivers but also provides a more natural and user-driven interaction with the assistive system.

While this study primarily focused on validating the system’s feasibility with healthy participants, we acknowledge that the sample size (N = 5) is limited, which restricts the generalizability of the results. Future research will address this by conducting experiments with a more diverse participant pool, including individuals with motor impairments and different age groups, to better assess usability and adaptability in real-world settings. Additionally, testing under varied environmental conditions, such as different lighting settings and user movement patterns, will further strengthen the robustness of the system for real-world deployment.

In terms of environmental factors, previous studies [54] showed that pupillary responses to stimuli are significantly reduced in environments with illuminance below 150-lux. In our experimental setup, the average illuminance level was 20-lux, which suggests that ambient lighting conditions had minimal impact on the measured PLR signals. However, considering that real-world environments may involve more dynamic lighting variations, future research should explore the effects of different illuminance levels on system performance to ensure robust applicability in practical scenarios.

Conducting comparative evaluations with other hybrid BCI-based assistive systems, such as SSVEP or motor imagery-based control methods, will also help contextualize the advantages and trade-offs of our approach. Improving adaptive automation through real-time behavioral adjustments and enhancing signal processing for greater classification robustness will be key areas of focus in optimizing the system for broader deployment. These developments will ensure that the system is not only practical for real-world assistive applications but also maintains strong clinical relevance and broader generalizability across diverse user needs.

## 5. Conclusions

Existing SSVEP-based BCI systems, including those used in meal-assist robots, are known for their high accuracy. However, the practical limitations of wet electrodes hinder their usability. This paper proposes a hybrid BCI system utilizing mobile EEG devices and PLR, which demonstrates high accuracy. By employing dry-type electrodes, this system is suitable for real-world applications. Furthermore, the integration of the PLR and EMG allows for an intuitive process from meal initiation to completion, enabling users to intentionally start eating, select their desired food, and repeat the cycle, as needed. This practical BCI-based meal-assist robot system has the potential to enhance the self-esteem of elderly and disabled individuals who rely on it.

## Figures and Tables

**Table 1:** Parameters of the 1D-TCN model.

Layer	Type	Shape (B, F, T) ^1^	Filter Size	Kernel Size	Dilations
Input	PLR-CWT	(32, 122, 139)			
0	DepthwiseConv2D	(32, 122, 139)		(3, 1)	
1	TCN-1	(32, 139, 512)	512	2	[1, 2, 4, 8]
2	TCN-2	(32, 139, 512)	512		
3	TCN-3	(32, 139, 64)	64		
4	MaxPool1D	(32, 4416)			
5	Dropout ^2^	(32, 4416)			
6	FC ^3^	(32, 4)			
7	Softmax	(32, 4)			
Optimizer	Adam				
Learning rate	0.001				
Loss function	Sparse categorical crossentropy + 0.7 × Center loss				

^1^ B represents batch size. F denotes the number of frequency components after applying the CWT to PLR from both eyes. T represents the number of time points. ^2^ Dropout rate: 0.5 ^3^ Fully-connected layer.

**Table 2:** Performance (Accuracy, ITR ^1^) according to experiments (SSVEP ^2^, PLR ^3^, FVEP ^4^, and Hybrid ^5^).

Experiment	Performance	S1	S2	S3	S4	S5	Average
SSVEP	Accuracy (%)	88.33	85.00	98.33	100	95.00	93.33
	ITR (bit/min)	17.27	15.37	24.68	26.67	21.79	21.16
PLR	Accuracy (%)	66.75	84.98	95	92.75	84.85	84.87
	ITR (bit/min)	7.41	15.35	21.79	20.13	15.28	15.99
FVEP	Accuracy (%)	71.67	77.41	71.67	80.93	86.30	77.59
	ITR (bit/min)	8.29	10.45	8.29	11.94	14.48	10.57
Hybrid	Accuracy (%)	74.00	84.09	97.78	97.72	89.36	88.59
	ITR (bit/min)	10.15	14.88	24.15	24.09	17.90	18.23

^1^ ITR: Information transfer rate. ^2^ SSVEP: Steady-state visual evoked potential. ^3^ PLR: Pupillary light reflex. ^4^ FVEP: Flash visual evoked potentials. ^5^ Hybrid: Flash visual evoked potential and pupillary light reflex.

## Data Availability

The raw data supporting the conclusions of this article will be made available by the authors, without undue reservation.

## Supplementary Materials

The following supporting information can be downloaded at: https://www.mdpi.com/article/10.3390/biomimetics10020118/s1, Figure S1: CWT-based PLR features of each LED stimuli. References [55,56,57,58,59] are cited in the Supplementary Materials; Figure S2: The overview of geodesic distance between SPD matrices from EEG on the Riemannian manifold.
