# Supplementary material for: Hybrid BCI for Meal-Assist Robot Using Dry-Type EEG and Pupillary Light Reflex
Source: Biomimetics (Basel). 2025 Feb 18;10(2):118. doi: 10.3390/biomimetics10020118 (PMC11853533; doi:10.3390/biomimetics10020118)
Supplement: Supplementary file 1 [file biomimetics-10-00118-s001.zip › biomimetics-3426893-supplementary.pdf]

Supplementary materials

Supplementary 1. Continuous wavelet transforms (CWT)-based binocular PLR features

The CWT of PLR signal f(t), is expressed [48] by

$$W(s,t) = \frac{1}{\sqrt{s}} \int_{-\infty}^{\infty} f(\tau)\,\psi\!\left(\frac{\tau - t}{s}\right) d\tau$$

where t is the time variable, s the scale, and ψ is the continuous wavelet function in both the time domain and the frequency domain. W(s,t) is the CWT coefficient along the time axis, which calculates the correlation between the wavelet and the PLR signals. We used the Morse wavelet as the mother wavelet to extract features through CWT in the frequency range of 0.5–1.75 Hz. Morse wavelets are defined [21] by

$$\psi_{\beta,\gamma} = \int_{-\infty}^{\infty} \psi_{\beta,\gamma}(t) e^{-i\omega t} = U(\omega) a_{\beta,\gamma} \omega^{\frac{P^2}{\gamma}} e^{-\omega^{\gamma}},$$

where $\psi_{\beta,\gamma}(t)$ is the time domain wavelet function, $U(\omega)$ is unit step function, $a_{\beta,\gamma}$ is normalizing constant. The parameters β and γ of the Morse wavelet control the wavelet form in the Fourier domain. Thus, the parameters were set to $\gamma = 3$, and $p^2 = 60$, with P^2 defined as $\beta\gamma$. Figure S1 shows CWT-based features of PLR signals for each LED stimulus using the aforementioned method.

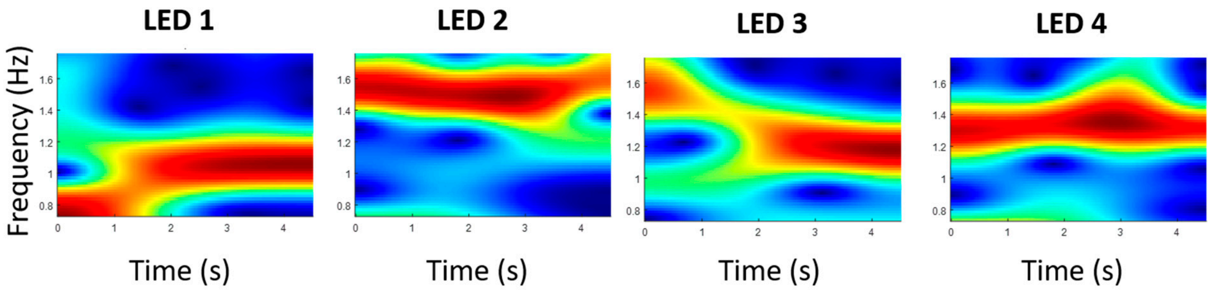

Figure S1. CWT-based PLR features of each LED stimulus

Supplementary 2. Symmetric Positive Definite (SPD) matrix and Riemannian geometry

For each trial of the experiment, segmented EEG data $X_i \in \mathrm{R}^{C\times N}$ was constructed,

$$\mathrm{X} = \begin{bmatrix} X_{C_1} \\ X_{C_2} \\ X_{C_3} \\ \vdots \\ X_{C_8} \end{bmatrix}$$

where C is the number of EEG channels and N is the temporal samples for length of a specific time window. The matrix $\Sigma \in \mathrm{R}^{C\times C}$ is SPD matrix, also referred as the covariance matrix, which contains the spatial information between the eight EEG channels (Fz, Pz, P4, P3, O1, O2, C3, and C4) in this analysis.

$$\Sigma = \frac{1}{N-1} XX^T$$

$$= \frac{1}{N-1} \begin{pmatrix} X_{C_1}{X_{C_1}}^T & \cdots & X_{C_1}{X_{C_8}}^T \\ \vdots & \ddots & \vdots \\ X_{C_8}{X_{C_1}}^T & \cdots & X_{C_8}{X_{C_8}}^T \end{pmatrix}$$

All SPD matrices generated from EEG data are deemed to form a differentiable Riemannian manifold M, which has a surface with non-positive curvature. To calculate the curve length between any two SPD matrices on the manifold M, the shortest path is called *geodesic*, which is illustrated in Figure S2 [44]. We referred to this as the Riemannian distance in the paper.

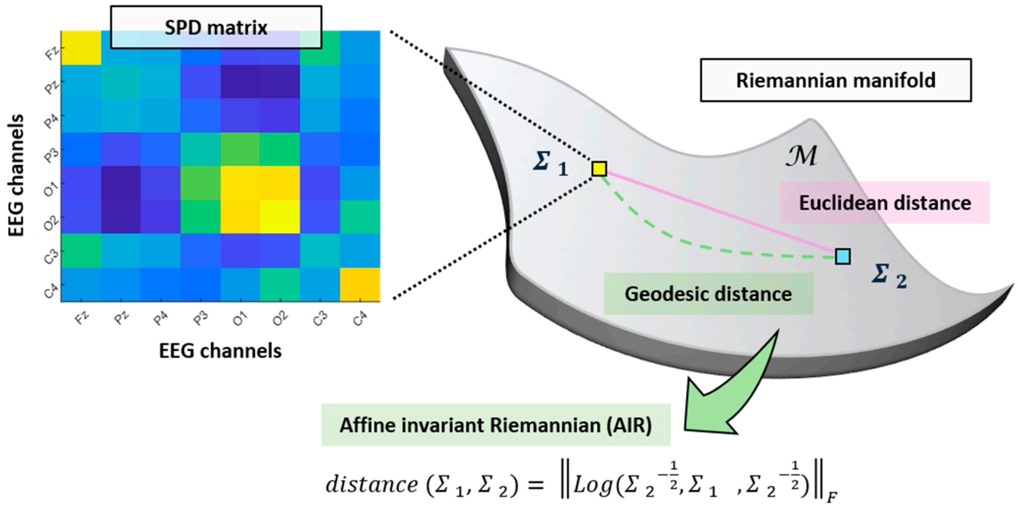

Figure S2. The overview of Geodesic Distance Between SPD Matrices from EEG on the Riemannian Manifold

We adopted affine invariant Riemannian (AIR), which transforms the Euclidean metric in vector spaces to the Riemannian manifold. This captures nonlinear associations, serves as a useful tool for decoding brain states, and leverages spatial information from EEG, which is commonly reported in BCI applications [45, 46, 47].

Supplementary References)

1. P. Petersen, Riemannian Geometry, Springer, 2006.

2. Xu, J.; Markham, A.; Meunier, A.; Raggam, P.; Grosse-Wentrup, M. Distance covariance: A nonlinear extension of Riemannian geometry for EEG-based brain-computer interfacing. In Proceedings of the 2021 IEEE International Conference on Systems, Man, and Cybernetics (SMC); IEEE, Melbourne, Australia, 2021; pp. 2000–2005. DOI: 10.1109/SMC52423.2021.9658876

3. Cichocki, A.; Cruces, S.; Amari, S.I. Log-determinant divergences revisited: Alpha-beta and gamma log-det divergences. En-tropy 2015, 17(5), 2988–3034. DOI: 10.3390/e17052988

4. Kalaganis, F.P.; Laskaris, N.A.; Oikonomou, V.P.; Nikopolopoulos, S.; & Kompatsiaris, I. Revisiting Riemannian geome-try-based EEG decoding through approximate joint diagonalization. J. Neural Eng 2022, 19, 066030. DOI:10.1088/1741-2552/aca4fc.

5. Samar, V. J.; Bopardikar, A.; Rao, R.; & Swartz, K. Wavelet analysis of neuroelectric waveforms: a conceptual tutorial. Brain and language 1999, 66(1), 7-60. DOI: https://doi.org/10.1006/brln.1998.2024
